# Physics‑informed machine learning modeling with dual SHAP analysis for predicting *Salmonella Enteritidis* growth on chicken meat treated with slightly acidic electrolyzed water

**DOI:** 10.1016/j.psj.2026.107507

**Published:** 2026-07-27

**Authors:** Yunxiang Zhu, Shan Bing, Yitian Zang, Yanjiao Li, Guoping Wu, Guosheng Zhang, Nengshui Ding, Guoyun Wu, Zhen Wang, Ruilin Wang, Fule Yang, Yucheng Zhang, Lili Xiao, Chenxi Cui, Yuhang Ma, Hongxin Fu

**Affiliations:** a Jiangxi Agricultural University, Jiangxi, 330045, China; b Jiangxi Agricultural Technology Extension Center, Jiangxi, 330046, China

**Keywords:** *Salmonella Enteritidis*, Slightly acidic electrolyzed water, Physics-informed machine learning, SHAP, Residual correction

## Abstract

Slightly acidic electrolyzed water (SAEW) is an effective sanitizer for controlling *Salmonella Enteritidis* in chicken processing, but existing mechanistic models suffer from systematic prediction bias due to structural simplifications, limiting its precise application under complex temperature scenarios. To address this bias, we developed a SGompertz–CatBoost residual-correction hybrid model and conducted dual SHAP analyses to diagnose bias sources and interpret decision mechanisms. The hybrid model achieved an R^2^ of 0.981 and reduced RMSE by 41.2% relative to the mechanistic model. Residual SHAP attributed the systematic bias to three structural deficiencies: the symmetric sigmoidal assumption, the linear temperature–available chlorine concentration (ACC) interaction, and the linear temperature response. Hybrid SHAP further verified that residual correction shifted the mechanistic prediction (*y_phys_*) from the primary bias source to a stable baseline; time–temperature interaction terms (*tT, tT^2^*, and *t^2^T*) autonomously captured nonlinear thermal kinetics; ACC of SAEW emerged as an independent antimicrobial signal; and the contributions of mechanistic parameters collapsed to near zero. Scenario-based prediction indicated that 30 mg/L ACC reduced predicted *S.* Enteritidis peaks by 1.5 – 1.9 log CFU/g across storage scenarios. Superiority probability heatmaps revealed that higher ACC SAEW consistently exhibited probabilistic dominance at 4 °C (0 – 350 h), 14 °C (0 – 130 h), and 24 °C (0 – 90 h). This study provides a robust framework for SAEW process optimization and risk assessment under controlled isothermal storage conditions.

## Introduction

*Salmonella Enteritidis* (*S.* Enteritidis) is a major cause of foodborne illness worldwide, with poultry products serving as a primary transmission route (Thames and Theradiyil Sukumaran, 2020). Temperature abuse during cold-chain storage can markedly accelerate its proliferation on chicken meat, thereby increasing both food safety risks and economic losses (Casanova et al., 2022; Li et al., 2017). To mitigate these risks, various decontamination strategies have been explored. Among them, slightly acidic electrolyzed water (SAEW) has gained increasing attention as an environmentally friendly sanitizer because of its broad antimicrobial spectrum, operational safety, and lack of harmful chemical residues (Yuan et al., 2023). SAEW is generated by electrolyzing a dilute NaCl or HCl solution in an electrolytic cell, producing free chlorine species—primarily hypochlorous acid (HClO)—as the main antimicrobial agent. With a pH of 5.0 – 6.5, SAEW exhibits strong bactericidal activity while being safe for human handling and leaving minimal chemical residues on food surfaces. Beyond its application in microbial decontamination of poultry products, SAEW has also been explored for improving meat quality attributes such as protein structure and gel properties during thawing and processing (Kong et al., 2022, 2023) .

However, the antimicrobial efficacy of SAEW depends on the interplay of multiple factors, including available chlorine concentration (ACC), pH, oxidation-reduction potential (ORP), treatment conditions, and subsequent storage temperature (Rahman et al., 2016; Yuan et al., 2023; Zang et al., 2024). This complexity makes it difficult to understand and predict the behavior of *S.* Enteritidis on SAEW‑treated chicken meat. As a result, there is a growing need for quantitative models capable of accurately describing microbial growth under such multifactorial conditions. To address this need, our previous work developed a tertiary SGompertz model that incorporates storage time, temperature, and ACC as explanatory variables to characterize *S.* Enteritidis growth after SAEW treatment (Zang et al., 2024). The model is parameterized by biologically interpretable quantities—maximum population density (*a*), maximum specific growth rate (*k*), and inflection time (*xc*)—and performs well under constant and controlled laboratory conditions.

Nevertheless, like many classical mechanistic models, the SGompertz formulation relies on simplifying assumptions that limit its predictive capability in real food systems. First, its symmetric sigmoidal structure does not reflect the asymmetric growth patterns often observed in food matrices, where extended lag phases or early stationary phases may arise due to environmental stress or resource limitation (Li et al., 2017; Zang et al., 2024). Second, its simplified temperature–ACC interaction fails to capture key phenomena such as the temperature‑dependent threshold behavior of SAEW disinfection and the repair kinetics of sublethally injured cells during storage (Liu et al., 2019; Rahman et al., 2016). These structural limitations lead to systematic prediction errors, particularly under low‑temperature storage, high ACC treatments, or prolonged storage durations. Correcting such systematic errors requires an approach that can learn from data while remaining grounded in biological principles. A hybrid modeling strategy—using a data‑driven model to learn the residual errors of a mechanistic baseline—offers a natural solution. Moreover, to ensure that the corrected model remains interpretable rather than becoming a “black box,” tools such as SHAP (SHapley Additive exPlanations) are essential for verifying the biological plausibility of the corrections and elucidating the complex interactions that the mechanistic model fails to capture.

To balance the interpretability of mechanistic models with the predictive power of machine learning, we propose a mechanistic model‑informed hybrid approach. In this framework, the SGompertz model serves as a knowledge‑guided baseline encoding biological prior information, while a CatBoost regressor functions as a residual learner to capture systematic deviations between model predictions and empirical observations. The final prediction is obtained by superimposing the learned residuals onto the mechanistic baseline, thereby combining interpretability with flexibility. The feasibility of such hybrid approaches has been demonstrated across diverse scientific fields. For example, Raissi et al. (2019) introduced physics‑informed neural networks (PINNs), showing that embedding physical constraints in machine learning models can substantially improve accuracy and generalization. More recently, Willard et al. (2023) highlighted residual learning guided by mechanistic outputs as one of the most effective paradigms for integrating scientific knowledge with data. This paradigm is particularly suitable for addressing the systematic biases arising from structural simplifications—such as symmetry assumptions and oversimplified factor interactions—in mechanistic models like SGompertz. However, no study to date has systematically applied this hybrid methodology to predict *S.* Enteritidis growth on SAEW‑treated chicken meat, nor integrated it with dual SHAP analysis to create a complete workflow from bias diagnosis to decision reconstruction.

Therefore, to overcome the limitations of purely mechanistic models and provide a more accurate and interpretable tool for predicting *S.* Enteritidis behavior, this study aimed to: (1) develop a hybrid predictive model combining a tertiary SGompertz formulation with CatBoost‑based residual correction to improve accuracy under complex conditions; (2) employ dual SHAP analysis to diagnose sources of systematic bias in the mechanistic model and to interpret the decision logic of the hybrid model; and (3) perform scenario‑based simulations to translate point predictions into comparative metrics, thereby identifying application windows with consistent antimicrobial advantages under representative isothermal cold‑chain storage scenarios (4, 14, and 24 °C) for chicken products.

## Materials and methods

### Bacterial strain and inoculum preparation

*S.* Enteritidis strain BNCC-103134 was obtained from the Bena Culture Collection Center (Henan, China). A rifampicin-resistant variant was generated following the procedure described by Zang et al. (2024) to enable selective enumeration in the presence of indigenous microflora. The strain was routinely maintained on tryptic soy agar (TSA) supplemented with rifampicin (100 mg/L; TSAr) at 4°C and subcultured at monthly intervals.

For inoculum preparation, a single colony was aseptically transferred into brain heart infusion (BHI) broth containing rifampicin (100 mg/L) and incubated at 37 °C for 18 – 20 h with agitation (150 rpm). Bacterial cells were harvested by centrifugation (5000 × g, 4 °C, 15 min), washed twice with sterile 0.1% peptone water (PW), and resuspended in PW to yield an inoculum of approximately 10^5^ CFU/mL.

### Preparation and characterization of SAEW

SAEW was generated using a non-membrane electrolysis system (Zhongshan Lady Li Electrical Co., Ltd., Guangdong, China) with an input voltage of 220 V. A total of 2 L of NaCl solution (0.5%, w/v) containing HCl (0.5 mg/L) was used as the electrolyte, and the electrolysis time was 30 min. Immediately after generation, SAEW was diluted with sterile deionized water to obtain working solutions with ACC of 10, 20, and 30 mg/L. The pH values of the diluted solutions were 6.03 ± 0.11, 6.12 ± 0.14, and 6.15 ± 0.15, and the ORP values were 822 ± 2, 834 ± 3, and 849 ± 2 mV for ACC of 10, 20, and 30 mg/L, respectively, measured prior to each experiment using a calibrated pH meter (Shanghai Leici Co., Ltd., Shanghai, China) and ORP meter (Shanghai Sanxin Co., Ltd., Shanghai, China), respectively. ACC was determined using a digital chlorine analyzer (Shanghai Haizheng Electronic Technology Co., Ltd., Shanghai, China).

### Chicken breast preparation, inoculation, and SAEW treatment

Fresh chicken breast meat was purchased from a local retail market and transported to the laboratory within 30 min under refrigerated conditions. Upon arrival, samples were aseptically trimmed to remove visible connective tissue and cut into 5.0 g portions (approximately 2.5 cm × 2.5 cm square pieces) using sterile surgical blades. Prior to inoculation, samples were confirmed to be *Salmonella*-negative by selective enrichment in Rappaport–Vassiliadis (RV) broth followed by plating on xylose lysine deoxycholate (XLD) agar. Background total viable counts (TVC) were determined by surface plating on TSA. Each meat portion was surface-inoculated with 100 μL of the prepared *S.* Enteritidis suspension (∼10^5^ CFU/mL), evenly distributed over the sample surface, and allowed to attach for 10 min in a biosafety cabinet. This resulted in an initial *S.* Enteritidis population of approximately 2.25 log CFU/g (to simulate realistic low-level contamination, Oscar, 2007) and an initial TVC of approximately 4.10 log CFU/g.

A total of 540 samples were randomly assigned to four experimental groups: an untreated control and three SAEW-treated groups with ACC values of 10, 20, and 30 mg/L (n = 135 per group). For SAEW treatment, inoculated samples were immersed in 1500 mL of the corresponding SAEW solution for 5 min (Al‑Holy and Rasco, 2015) , immediately transferred to 0.5% sodium thiosulfate solution for 6 s to neutralize residual chlorine, and then air-dried for 10 min.

Following treatment, each sample was individually packaged in UV-sterilized transparent polyethylene bags and stored in programmable temperature–humidity chambers (Shangcheng Biotechnology Co., Zhejiang, China) set at 4, 14, or 24 °C with 70% relative humidity. Sampling intervals were temperature-dependent (every 24 h at 4 °C, every 12 h at 14 °C, and every 8 h at 24 °C). At each sampling point, three samples per group were randomly selected for microbiological analysis.

### Microbiological analysis

Each sample was aseptically transferred into a sterile stomacher bag containing 90 mL of buffered peptone water and homogenized for 3 min using a laboratory stomacher (Shanghai Guanshen Biosafety Technology Co., Ltd., Shanghai, China). Homogenates were serially diluted ten-fold in buffered peptone water, and 0.1 mL aliquots of appropriate dilutions were surface-plated onto TSA and TSAr. All plates were incubated at 37 °C for 24 h. Colonies recovered on TSAr were enumerated as rifampicin-resistant *S.* Enteritidis. TVC were calculated as the difference between counts on TSA and TSAr, thereby representing the background microflora. Microbial populations were expressed as log CFU/g.

### Development of the hybrid predictive model

#### Mechanistic baseline model

A three-parameter SGompertz model developed in our previous study (Zang et al., 2024) was used as the mechanistic baseline. The model predicts *S.* Enteritidis population density (y_phys_) as a function of storage time (*t*), temperature (*T*), and ACC, with biologically interpretable parameters including the asymptotic maximum population (*a*), maximum specific growth rate (*k*), and inflection point (*xc*).

(1) $$\begin{array}{c} y=\left(a_{0}+b*\text{ACC}\right)*exp(-exp(\\ -\left(\left(k_{0}+n*T\right)+m*T*\text{ACC}\right)*\left(t-\left(x_{c0}+i*T+j*ACC+g*ACC*T\right)\right))) \end{array}\;$$

#### CatBoost-based residual learning

The residual term was defined as the difference between the observed bacterial count and the mechanistic prediction:

(2) $$r=y_{\text{obs}}-y_{\text{phys}}$$

In Equation (2), r represents the residual, defined as the difference between the observed microbial count and the value predicted by the mechanistic model. The term y_obs_ refers to the experimentally measured microbial count, expressed in log CFU per gram. The term y_phys_ denotes the microbial count predicted by the mechanistic model under the same conditions.

A CatBoost regression algorithm was employed to model this residual. The input feature set comprised: (*i*) primary predictors (*t, T*, ACC); (*ii*) nonlinear transformations *log (1+t), t^2^, T^2^*, and ACC*^2^*; (*iii*) interaction terms (*tT, t⋅*ACC, *T⋅*ACC, *t^2^T, tT^2^, t^2^⋅*ACC); and (*iv*) physics-informed variables, including y_phys_ and the mechanistic parameters (*a, k, xc *). *T*ACC: Cumulative interaction term between temperature and ACC, defined as the product of these two variables. *k_phys_*: Growth rate constant estimated from the mechanistic model, expressed per hour. *xc*: Inflection time estimated from the mechanistic model, expressed in hours. *tT*: First‑order interaction term between time and temperature, defined as the product of time and temperature. *tT^2^*: Interaction term between time and the squared temperature, defined as time multiplied by the square of temperature. *t^2^T*: Interaction term between the squared time and temperature, defined as the square of time multiplied by temperature. *tC^2^*: Interaction term between time and the squared available chlorine concentration, defined as time multiplied by the square of the available chlorine concentration. *t^2^*: Squared time term. ACC*^2^*: Squared available chlorine concentration term.

The entire dataset (n = 540) was randomly partitioned into a training set (80%, n = 432) and a hold‑out test set (20%, n = 108), stratified by temperature (4, 14, and 24°C) to ensure that all storage conditions were proportionally represented in both subsets. The training set was used for model development and hyperparameter tuning via five‑fold cross‑validation, while the hold‑out test set was strictly reserved for final performance evaluation and was not exposed to the model during any stage of training or parameter selection. To prevent overfitting, CatBoost’s built‑in ordered boosting mechanism and an early‑stopping strategy based on validation set performance were applied during cross‑validation. Model hyperparameters were optimized using five-fold cross-validation. The final hybrid prediction was obtained as:

(3) $$\hat{y}=y_{\text{phys}}+\hat{r}$$

In this equation, ŷ represents the final predicted microbial count produced by the hybrid model. The term r̂ represents the estimated value of the residual r, which was defined in Equation (3), and is predicted by the data‑driven component of the hybrid model. By incorporating the estimated residual, the hybrid model corrects systematic deviations in the mechanistic predictions.

### Model interpretation using SHAP

Model interpretability was achieved using SHAP. For the residual model, SHAP values quantified the contribution of individual features to the prediction error of the mechanistic model, enabling identification of systematic deviations. For the hybrid model, SHAP analysis elucidated the relative importance and directionality of feature effects on the final predicted bacterial counts. Beeswarm plots, summary bar plots, and waterfall plots were generated using the Python shap library.

### Scenario simulation

Scenario-based simulations were conducted to compare the probabilistic behavior of predicted *Salmonella* concentrations under different SAEW treatments during under constant‑temperature storage conditions representative of cold‑chain scenarios. Rather than introducing any absolute microbial threshold, treatment effects were quantified using a pairwise probabilistic comparison framework.

Specifically, for a given storage scenario, the treatment effect was defined as the probability that the predicted *Salmonella* concentration under higher-ACC SAEW was lower than that under lower -ACC or control treatments:

(4) Δy=yACC=10−yACC=30

Here, *y* is treated as a random variable drawn from the predictive distribution generated by repeated stochastic simulations of the hybrid model under identical (*T, t*, ACC) conditions. The advantage probability was defined as:

(5) P(T,t)=Pr⁡[yACC=30<yACC=10]

In Equation (4), Δy represents the treatment effect, defined as the difference between the predicted responses under ACC = 10 mg/L and ACC = 30 mg/L. In Equation (5), *P*(*T, t*) denotes the advantage probability, defined as the probability that the predicted response under ACC = 30 mg/L is lower than that under ACC = 10 mg/L at treatment condition *T* and time *t*. An advantage probability threshold of 0.8 was used to identify regions where SAEW consistently outperformed the control.

### Statistical analysis

Microbiological data are presented as mean ± standard deviation. Each storage condition consisted of three independent biological replicates, with two technical replicates per biological replicate. Statistical differences among treatments were assessed by one-way analysis of variance (ANOVA) followed by Duncan’s multiple range test using SPSS 26.0 (SPSS Inc., Chicago, IL, USA). Differences were considered statistically significant at *P* < 0.05.

Model performance was evaluated using the coefficient of determination (R^2^), root mean square error (RMSE), and mean absolute error (MAE). All data processing, statistical analyses, and graphical visualization were conducted using Python 3.8 (NumPy, Pandas, Scikit-learn, CatBoost, SHAP and Openpyxl).

## Results and discussion

### Comparison of predictive accuracy among the mechanistic, pure machine learning, and hybrid models

**Fig. 1:**
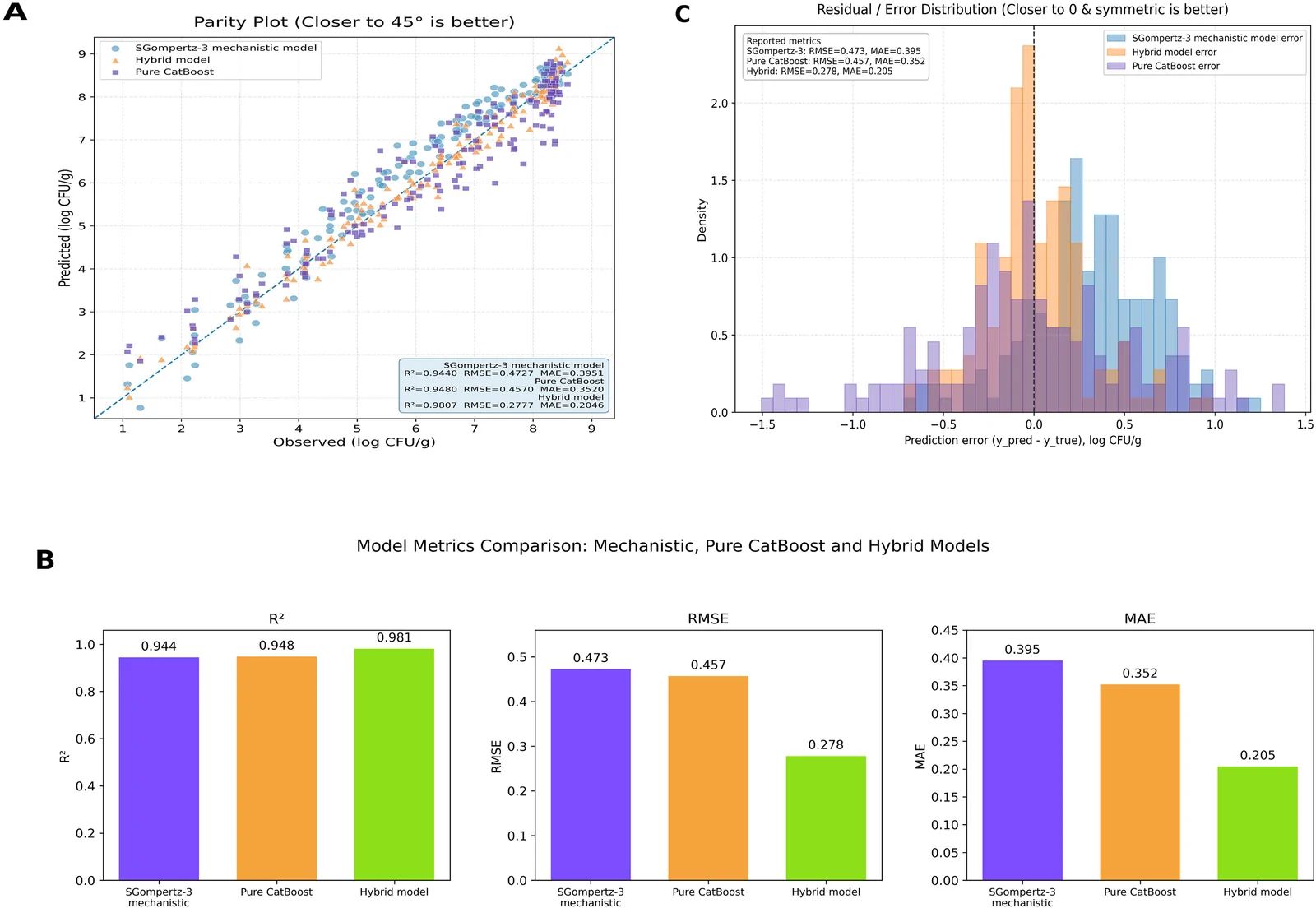
**Comparison of predictive performance among the SGompertz-3 mechanistic model, pure CatBoost model, and hybrid model.** Note: (A) Scatter plots comparing the observed and predicted bacterial counts in the test set. Predictions from the SGompertz-3 mechanistic model (blue circles), hybrid model (orange triangles), and pure CatBoost model (purple squares) are shown. The diagonal dashed line represents the one-to-one reference. (B) Comparison of model performance metrics, including the coefficient of determination (R²), root mean square error (RMSE), and mean absolute error (MAE), among the three models. (C) Distributions of prediction errors (predicted minus observed) for the SGompertz-3 mechanistic model (blue), hybrid model (orange), and pure CatBoost model (purple). The black dashed line indicates zero prediction error.

To evaluate the predictive performance of the hybrid model, its results on the test set were compared with those of the pure SGompertz mechanistic model and a purely data‑driven CatBoost benchmark trained directly on the raw input features (*t, T,* ACC) without any physics‑based information. As shown in Fig. 1A, predictions from the mechanistic model alone (blue) deviate systematically from the identity line. In the mid-to-high range (5 – 9 log CFU/g), clear overestimation is observed, whereas predictions in the low range (1 – 3 log CFU/g) exhibit substantial dispersion. In contrast, predictions from the hybrid model (orange) are tightly clustered around the identity line, with markedly reduced scatter.

The quantitative metrics in Fig. 1B further corroborate these visual differences. The hybrid model achieved an R^2^ of 0.981, an RMSE of 0.278 log CFU/g, and an MAE of 0.205 log CFU/g, whereas the corresponding values for the pure mechanistic model were 0.944, 0.473, and 0.395 log CFU/g, respectively. Relative to the mechanistic model, the hybrid approach reduced RMSE by 41.2% and MAE by 48.1%. To further justify the physics‑informed design, we compared the hybrid model against a pure ML benchmark—a stand‑alone CatBoost model trained directly on the raw input features (*t, T,* ACC) without the SGompertz baseline or any physics‑derived interaction terms, using the same training/test split and five‑fold cross‑validation procedure. As summarized, the pure ML model achieved an R² of 0.948, an RMSE of 0.457 log CFU/g, and an MAE of 0.352 log CFU/g on the test set. The hybrid model substantially outperformed the pure ML benchmark, reducing RMSE by 17.9% and MAE by 14.7%. These results confirm that incorporating the SGompertz mechanistic baseline provides a quantifiable improvement over purely data‑driven modeling, validating the “physics‑informed” strategy adopted in this study. This level of accuracy also surpasses that reported in recent comparable studies (RMSE = 0.3 – 0.6 log CFU/g), demonstrating that residual correction can substantially enhance the predictive performance of mechanistic models.

Fig. 1C presents the density distributions of prediction residuals for both models. The residuals of the mechanistic model (blue) are clearly right-skewed, with a mean of + 0.25 log CFU/g and a wide range from − 0.75 to + 1.25 log CFU/g. In contrast, residuals from the hybrid model (orange) approximate a normal distribution centered near zero (− 0.05 to + 0.05), with a narrower range (− 0.5 to + 0.25 log CFU/g). The near-unbiased and concentrated residual distribution indicates that systematic, storage-condition–dependent error components have largely been eliminated.

Overall, the hybrid modeling strategy—using a three-parameter SGompertz model as the mechanistic baseline and CatBoost as a residual corrector—substantially improves the accuracy and robustness of *S.* Enteritidis count predictions while fully preserving the interpretable parameter structure of the original model. The sources of the remaining systematic bias require further diagnosis through residual distributions and feature attribution; this issue is addressed in Section 3.2 using SHAP analysis.

### Feature attribution of prediction bias in the mechanistic model: residual SHAP analysis

**Fig. 2:**
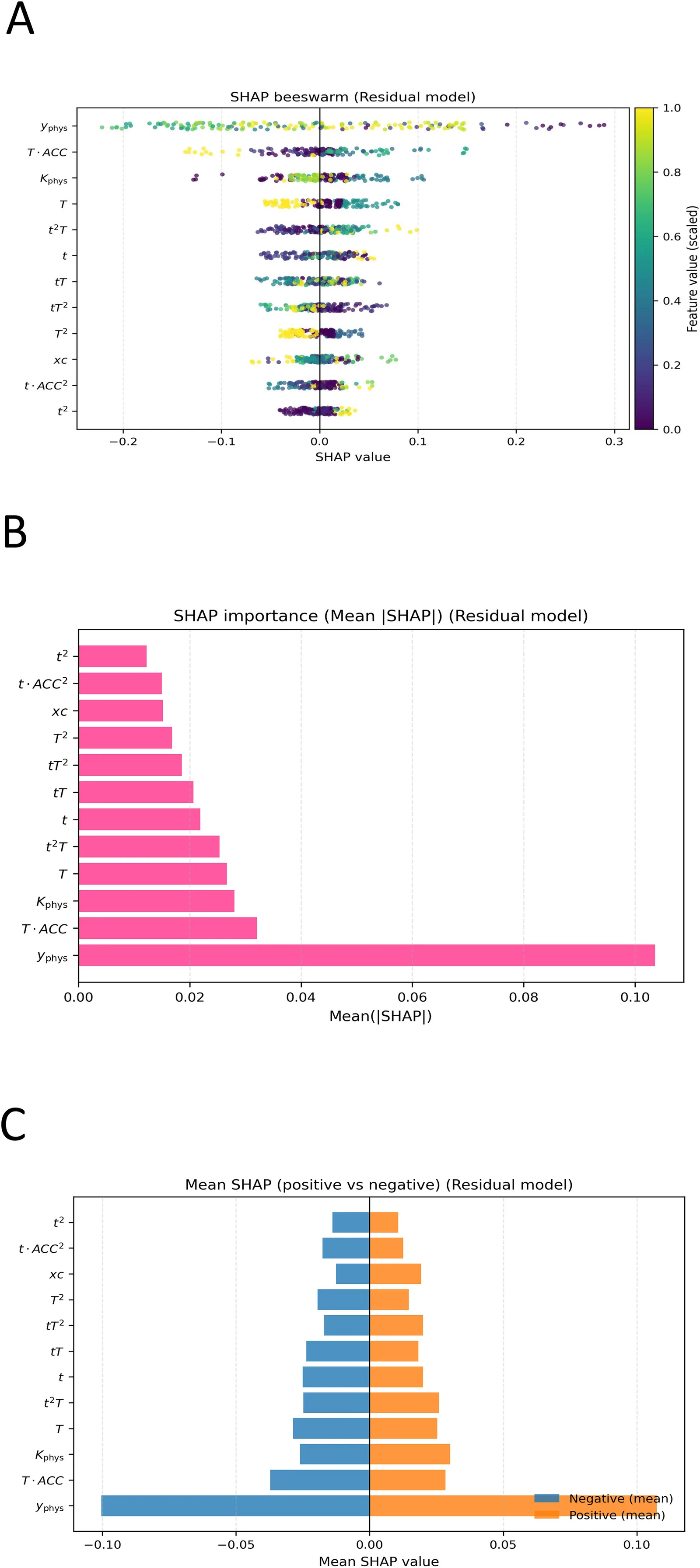
**SHAP analysis of the residual model: feature attribution of mechanistic model prediction bias.** Note: (A) SHAP beeswarm plot. Distribution of feature contributions to the predicted residuals of the mechanistic model. Each point represents one sample, with color indicating the magnitude of the feature value from low (purple) to high (yellow). The x‑axis represents the SHAP value: positive SHAP values indicate that a feature pushes the mechanistic model toward underestimation, while negative values indicate overestimation. Features are ordered by their average absolute SHAP values. (B) Feature importance bar plot. Average absolute SHAP values quantifying each feature’s overall contribution to correcting the mechanistic model’s bias. (C) Bidirectional contribution bar plot. Mean positive and negative SHAP contributions of each feature to the residual prediction, shown on the right (orange) and left (blue) sides respectively.

To investigate the origins of the systematic overestimation exhibited by the mechanistic model, a global SHAP interpretation was performed on the CatBoost residual model (Fig. 2). Fig. 2A shows the SHAP beeswarm plot, Fig. 2B presents mean |SHAP| values, and Fig. 2C compares positive and negative contribution magnitudes. Features are discussed below in descending order of importance.

To interpret the SHAP results presented below, the SHAP value on the x‑axis quantifies each feature's contribution to the model's prediction: positive values indicate that the feature pushes the prediction upward, while negative values indicate a downward effect. In the beeswarm plot (Fig. 2A), the color scale (purple to yellow) represents the feature value from low to high, allowing visualization of how the magnitude of each input variable influences its contribution.

#### Mechanistic model output (y_phys_): conflict between the symmetric sigmoidal assumption and asymmetric growth

As shown in Fig. 2A, y_phys_ exhibits the widest SHAP value range (− 0.2 to + 0.3), with a mean |SHAP| of 0.105, accounting for approximately 40% of the total contribution. Its positive (+ 0.11) and negative (− 0.10) contributions are comparable in magnitude, forming a balanced bidirectional pattern: the model tends to exhibit high uncertainty at low predicted values and overestimate at high predicted values. This bias originates from the symmetric sigmoidal assumption inherent in the SGompertz model. In reality, *S.* Enteritidis growth curves in food matrices are often asymmetric: environmental stress may prolong the lag phase, leading to underestimation, whereas resource limitation or inhibitory factors may induce an earlier stationary phase, resulting in overestimation (Paganini et al., 2022). Jia et al. (2020) further demonstrated, using competitive growth models, that background microbiota suppress the maximum population density of *Salmonella*, causing the stationary phase to occur at levels well below theoretical predictions under axenic conditions. By enforcing a fixed inflection point and symmetric curvature across all conditions, the mechanistic model inevitably produces opposite-direction biases at low and high prediction ranges, fully consistent with the scatter patterns observed in Section 3.1.

#### Temperature–ACC interaction (TACC): linear simplification versus nonlinear synergistic inhibition

In Fig. 2A, *T*ACC spans SHAP values from − 0.15 to + 0.15, with a mean |SHAP| of 0.033. Its negative contribution (− 0.04) is notably larger than its positive contribution (+ 0.03), indicating systematic overestimation under high *T*ACC conditions and underestimation under moderate *T*ACC conditions. This asymmetry arises from the mechanistic model’s use of a linear function to describe the interaction between temperature and SAEW concentration. However, the antimicrobial activity of SAEW exhibits pronounced temperature-dependent threshold behavior: synergistic inhibition is nonlinearly amplified at low temperatures (4 – 8°C), peaks at moderate temperatures (∼14°C), and shows diminishing marginal returns at higher temperatures (Rahman et al., 2016; Zang et al., 2019). Liu et al. (2019) further demonstrated via atomic force microscopy that SAEW treatment induces extensive sublethal cellular damage, whose repair may take hours to days. By fitting this inherently nonlinear interaction with a single linear term and neglecting sublethal injury–repair dynamics, the mechanistic model necessarily introduces systematic, directionally biased prediction errors across the *T*ACC range.

#### Mechanistic growth-rate constant (k_phys_): linear temperature response and missing interaction with background competition

As shown in Fig. 2A, SHAP values for k_phys_ range from − 0.15 to + 0.10, with a mean |SHAP| of 0.029. Positive contributions (+ 0.035) slightly exceed negative contributions (− 0.025), indicating a slight tendency toward underestimation, while still exhibiting substantial bidirectional bias.

This bidirectional pattern can be explained by the linear simplification of temperature effects combined with the omission of interactions with background microbial competition. As a mesophilic organism, *S.* Enteritidis experiences strong competitive suppression by background microbiota—particularly psychrotrophs—at low temperatures (4 – 8°C), resulting in apparent growth rates far below those predicted by linear temperature extrapolation (Jia et al., 2020; Odeyemi et al., 2020). When background loads are high, the model overestimates growth (negative residuals); when they are low, underestimation dominates (positive residuals). Because the mechanistic model employs a single linear temperature function and excludes batch-level heterogeneity due to background competition, these opposing effects coexist in the SHAP distribution.

#### Temperature and time–temperature interaction terms: dual structural deficiencies in temperature kinetics and inflection-point assumptions

Fig. 2A shows that SHAP values for the temperature main effect (*T*) range from − 0.05 to + 0.10, while time–temperature interaction terms (*tT, tT^2^, t^2^T*) are centered around zero with approximately symmetric contributions (± 0.02 – 0.03). Their mean |SHAP| values (0.020 – 0.026) are far lower than those of y_phys_ and *T*ACC. Although individually less influential, their joint patterns reveal two structural deficiencies in the mechanistic temperature formulation.

First, the linear assumption for the temperature dependence of growth rate is inconsistent with the true S-shaped response. The growth rate of *S.* Enteritidis follows an S-shaped thermal response (Guillén et al., 2024): the concave lower segment at 4 – 8°C, an accelerating region around 14°C, and diminishing marginal gains near 24°C. A linear approximation therefore overestimates growth at low temperatures, underestimates it at moderate temperatures, and overestimates it again at higher temperatures—exactly matching the SHAP patterns observed for temperature-related features (Guillén et al., 2024).

Second, the linear formulation of the inflection-point location fails to capture nonlinear transitions in growth initiation. The mechanistic model defines the inflection point *xc* as a linear function of temperature, implicitly assuming that the transition from lag to exponential phase shifts smoothly along the temperature axis (Baek et al., 2024; Gibson et al., 1987). In reality, growth initiation is governed by stochastic processes such as injury repair and competitive release, producing threshold-like transitions: prolonged stagnation at 4°C, abrupt shortening of lag time around 14°C, and diminishing marginal change near 24°C. The linear inflection-point function cannot accommodate this nonlinearity, leading to systematic underestimation of growth progression at mid-to-high temperatures—manifested as positive residual contributions from time–temperature interaction terms under warm, long-duration conditions.

Concentration-related features (*xc, tC^2^*) and higher-order time terms (*t^2^*) have mean |SHAP| values below 0.018, with small and symmetric contributions (< ± 0.02), indicating that they are not primary sources of systematic bias and only provide marginal adjustments.

Overall, residual SHAP analysis attributes the mechanistic model’s systematic prediction bias to three structural deficiencies: (1) the conflict between symmetric sigmoidal assumptions and asymmetric growth; (2) linear simplification of the temperature–ACC interaction, neglecting nonlinear synergy and sublethal injury repair; and (3) deficiencies in temperature kinetics, including linear temperature dependence of growth rate and linear inflection-point functions. These findings clearly identify the targets addressed by residual correction in the hybrid model.

### Feature contributions to hybrid model predictions: hybrid SHAP analysis

To elucidate how the residual-corrected model integrates features to generate final predictions, a global SHAP analysis was performed on the hybrid model.

#### Reconstruction of feature importance: from bias sources to predictive baseline

**Fig. 3:**
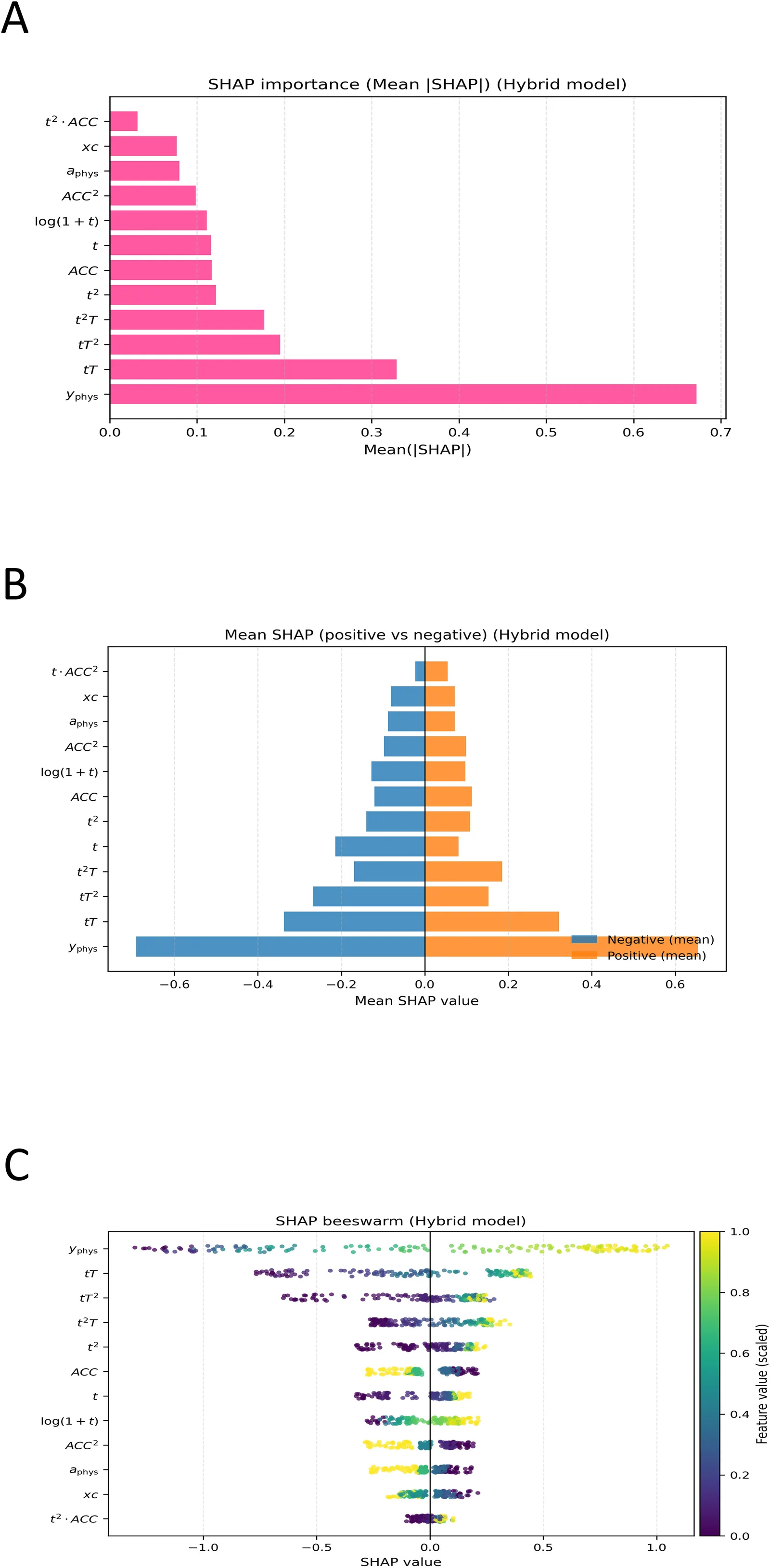
**SHAP analysis of the hybrid model: feature contribution to the final prediction.** Note: (A) Feature importance bar plot. Average absolute SHAP values ranking the contribution of each feature to the final predicted microbial count. (B) Bidirectional contribution bar plot. Mean positive (orange) and negative (blue) contributions of each feature to the final prediction. (C) Waterfall plot of a representative sample. Decomposition of the predicted microbial count into contributions from individual features. Yellow bars show features that increase the prediction, and blue bars show features that decrease it. The x‑axis represents the SHAP value, where the magnitude indicates the strength of each feature's contribution to the final predicted microbial count.

As shown in Fig. 3A, feature importance in the hybrid model exhibits a clear hierarchical structure. y_phys_ overwhelmingly dominates, with a mean |SHAP| of 0.67—twice that of the second-ranked feature (*tT*, 0.33)—accounting for ∼30% of the total contribution. This indicates that CatBoost-based residual correction successfully absorbs systematic bias, allowing the corrected mechanistic output to re-emerge as a reliable predictive backbone (with balanced positive and negative contributions in Fig. 3B). Compared with the three-parameter SGompertz model reported by Zang et al. (2024), this highlights a key distinction: in a purely mechanistic framework, y_phys_ is the final output and its bias cannot self-correct, whereas in the hybrid model it serves as a calibrated starting point upon which residual correction operates. SHAP comparisons thus demonstrate that residual learning reconciles physical interpretability with high predictive accuracy.

#### tT, tT^2^, and t^2^T: core correction terms compensating for missing nonlinear kinetics

The mean |SHAP| values of *tT, tT^2^*, and *t^2^T* are 0.33, 0.20, and 0.18, ranking second to fourth and exceeding their counterparts in the residual SHAP analysis (all < 0.025) by more than an order of magnitude. As shown in Fig. 3B, *tT* exhibits highly balanced contributions (− 0.30 / + 0.32), suggesting an approximately linear influence; *tT^2^* shows slightly stronger negative contributions (− 0.23 / + 0.16), exerting a stronger downward adjustment at low values; and *t^2^T* displays dominant positive contributions (− 0.14 / + 0.18), substantially elevating predictions at high values.

Together, these features indicate that the hybrid model autonomously captures nonlinear promotional effects of prolonged exposure at elevated temperatures, with interaction terms of different orders fulfilling distinct compensatory roles. As established in Section 3.2, the mechanistic model’s linear temperature response and linear inflection-point assumptions fail to describe nonlinear acceleration in growth rate and initiation kinetics. The prominent roles of *tT, tT^2^*, and *t^2^T* thus represent systematic, data-driven compensation: *tT* corrects first-order linear interactions, *tT^2^* captures higher-order temperature nonlinearity, and *t^2^T* accounts for coupling between nonlinear time effects and temperature.

Compared with previous models, this study achieves explicit separation of interaction orders in *S.* Enteritidis prediction on chicken surfaces. Traditional three-parameter SGompertz models compress temperature–ACC interactions into a single linear term, obscuring differences between “high-temperature short-time” and “moderate-temperature long-time” scenarios (Zang et al., 2024). Although Guillén et al. (2024) reveals S-shaped temperature dependence, its parameterization relies largely on linear extrapolation and systematically overestimates growth at low temperatures (Guillén et al., 2024). The hybrid model, through *tT, tT^2^*, and *t^2^T* contributions, provides a data-driven correction to these linear assumptions. Moreover, Jia et al. (2020) reported a competitive crossover temperature of 16.8°C, above which *Salmonella* gains a growth advantage over background microbiota. The strong positive contributions of *tT* under high-temperature, long-duration conditions in this study align precisely with that crossover, indicating that the model autonomously captures temperature-dependent competitive dynamics.

#### ACC: an independent antimicrobial signal

ACC has a mean |SHAP| of 0.11, comparable to the main time effect (*t*) and logarithmic time (*log (1+t)*) (Fig. 3A). In Fig. 3B, its contributions are approximately symmetric (± 0.09), with high ACC corresponding to negative contributions and low ACC to positive ones. Importantly, this contribution is independent of time–temperature interaction terms (interaction values between ACC and *tT* < 0.003 in Fig. 3A), indicating that ACC functions as an independent indicator of SAEW antimicrobial strength. This finding closes the loop with the residual diagnosis in Section 3.2: the mechanistic model systematically overestimates long-term SAEW inhibition due to linear interaction assumptions and omission of sublethal injury repair, whereas the hybrid model compensates for this deficiency through the negative main effect of ACC.

Substantial biological evidence supports the independent antimicrobial role of ACC. Yuan et al. (2023) observed pronounced membrane perforation in *Salmonella* cells after low-concentration electrolyzed water treatment using SEM, confirming extensive sublethal injury requiring hours to days for repair. Xuan et al. (2017) reported that sublethal stress primarily prolongs the lag phase of Listeria monocytogenes with minimal effect on growth rate. The predominately negative, time-independent SHAP contributions of ACC in this study are highly consistent with these observations. Possas et al. (2016) further noted that natural antimicrobials exert effects not only through initial lethality but also via sustained inhibition throughout storage. In contrast, the mechanistic model adjusts only the initial population, ignoring injury–repair dynamics and persistent inhibition. By incorporating ACC as an independent main effect, the hybrid model provides a data-driven correction for these mechanisms. Notably, ACC is the only feature directly representing intervention intensity, enabling quantitative assessment of microbial reduction across concentrations.

#### t and log (1+t): adaptive reconstruction of the time coordinate

The mean |SHAP| of t is 0.11, but its negative contributions (− 0.22) substantially exceed its positive contributions (+ 0.08), indicating that short durations exert a much stronger suppressive effect than long durations exert a promotional one. In contrast, *log (1+t)* has the same mean |SHAP| (0.11) but balanced bidirectional contributions (± 0.09).

This contrast indicates that the hybrid model no longer strictly adheres to the arithmetic time coordinate assumed by mechanistic models, but instead adaptively reconstructs temporal perception through feature engineering. As discussed in Section 3.2.1, microbial growth curves in food matrices are often asymmetric, with prolonged lag phases and earlier-than-expected stationary phases. The mechanistic model enforces symmetric curvature and fixed inflection points, inevitably producing opposite-direction biases. Logarithmic time transformation stretches the lag phase and compresses the stationary phase, enabling more flexible fitting of asymmetric growth, while the dominance of negative contributions from *t* reinforces suppression during early growth stages. This behavior represents a clear shift from “physical formula extrapolation” toward “data-driven calibration.”

The modified Gompertz model proposed by Gibson et al. (1987) remains a cornerstone of predictive microbiology, yet its arithmetic time axis implicitly assumes uniform progression along calendar time. The comparable importance of *log (1+t)* and *t*, combined with their contrasting contribution asymmetries, demonstrates that the hybrid model has adaptively redefined temporal representation, providing an important correction to the rigid temporal assumptions of classical models.

#### a, xc, and a_phys_: effective absorption of physical parameter bias

The mean |SHAP| values of *xc* is 0.08, and that of *a_phys_* is 0.09, placing them at the bottom of the importance ranking (Fig. 3A). Their contribution magnitudes are small (± 0.02 – 0.05) and largely symmetric (Fig. 3B), in stark contrast to their prominence as bias sources in the residual SHAP analysis (Section 3.2). This reversal constitutes decisive evidence of effective residual correction: CatBoost has successfully learned and compensated for systematic deficiencies in the mechanistic estimates of maximum population density (*a*), inflection-point location (*xc*), rendering the hybrid model no longer dependent on these biased parameters.

This result aligns with recent advances in physics-informed machine learning for biological process modeling, where prior physical knowledge serves as informative feature engineering while its parametric form is subject to posterior, data-driven correction (Raissi et al., 2019; Willard et al., 2023). In particular, Raissi et al. (2019) emphasized embedding physical knowledge as constraints or inputs within data-driven frameworks. Here, we adopt this paradigm in predictive microbiology by using SGompertz outputs as features and applying CatBoost-based residual learning to absorb bias. Willard et al. (2023) reviewed multiple physics-guided machine learning strategies and identified the use of physical model outputs as machine learning inputs as one of the most direct and effective approaches. This study operationalizes that paradigm and quantitatively demonstrates how physical knowledge can be post hoc calibrated by data.

The squared concentration term (ACC*^2^*) and the time–concentration interaction (*t^2^C*) exhibit mean |SHAP| values of 0.10 and 0.03, respectively, with weak and symmetric contributions, providing only minor saturation-level adjustments under high-concentration conditions.

#### Single-sample waterfall plot: microscopic validation of decision logic

Fig. 3C presents a SHAP waterfall plot for a representative sample. The contribution decomposition clearly illustrates the hybrid model’s decision chain: y_phys_ establishes a high baseline; *tT^2^* and *tT* indicate strong positive effects under high-temperature, long-duration conditions; *t^2^* reflects saturation as growth exceeds the optimal window, consistent with stationary-phase theory (Paganini et al., 2022); ACC and ACC*^2^* indicate that moderate ACC and concentration still permit some growth potential; *log (1+t)* and *t^2^T* constrain excessive growth; and physical parameters (a_phys_, *xc*) provide minor fine-tuning. As proposed by Li et al. (2025), SHAP not only quantifies global feature importance but also decomposes individual predictions. Fig. 3C demonstrates a coherent decision logic—“mechanistic baseline, temperature–time interaction as core correction, ACC as independent modulation, and temporal and higher-order terms as fine adjustments”—fully consistent with the global SHAP results, thereby validating the robustness and interpretability of the hybrid model.

### Prediction of S. enteritidis counts and evaluation of SAEW treatment effects under different storage scenarios

While SHAP analysis reveals ACC as an independent antimicrobial signal, it does not directly quantify risk reduction under different storage conditions or identify optimal concentration–temperature combinations. This section applies the validated hybrid model to multi-scenario simulations to systematically evaluate the effective operational window of SAEW.

#### Quantification of SAEW treatment effects

**Fig. 4:**
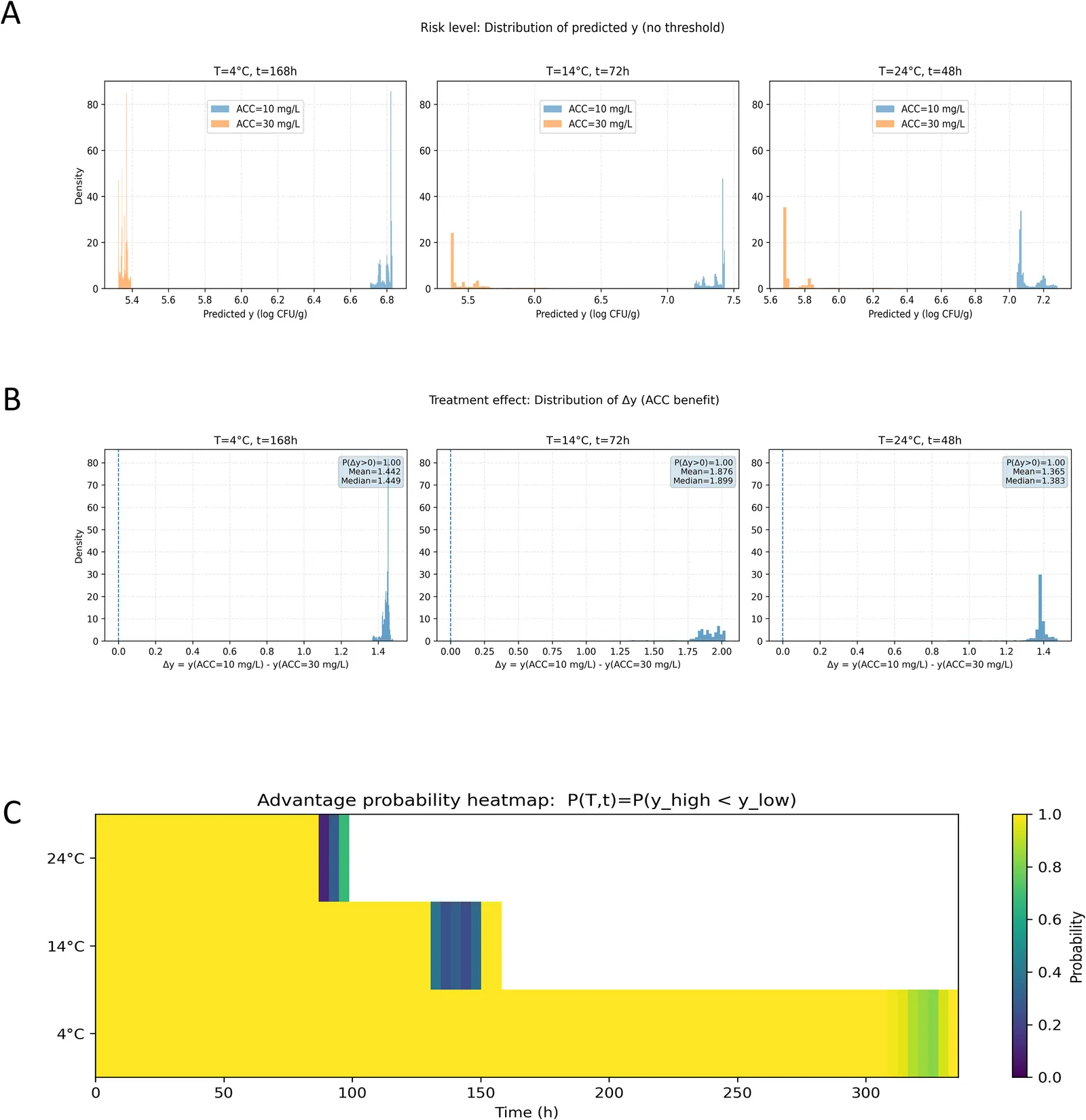
**Scenario‑based simulation of SAEW treatment effects and probabilistic superiority across storage conditions.** Note: (A) Predicted distributional shifts of *Salmonella* Enteritidis counts under representative storage scenarios. Probability density distributions of hybrid‑model predictions at 4 °C for 168 h, 14 °C for 72 h, and 24 °C for 48 h, comparing SAEW treatments with available chlorine concentrations (ACC) of 10 mg/L and 30 mg/L. (B) Probability density distributions of Δy=yACC=10−yACC=30 at 4, 14, and 24 °C. Boxplots indicate the mean, median, and interquartile range. (C) Advantage probability heatmap. Probability that the 30 mg/L treatment results in a lower microbial level compared with the 10 mg/L treatment, across temperatures (4, 14, and 24 °C) and storage times from 0 to 300 h. Colors approaching yellow indicate higher confidence in the antimicrobial advantage of the higher treatment concentration. An advantage probability threshold of 0.8 was used to define high‑confidence regions.

To quantify the antimicrobial efficacy of SAEW under different storage conditions, three representative scenarios were selected: 4°C for 168 h (end of conventional cold chain), 14°C for 72 h (short-term temperature abuse), and 24°C for 48 h (severe abuse). Fig. 4A shows the predicted probability density distributions of *S.* Enteritidis counts at ACC = 10 and 30 mg/L for each scenario, while Fig. 4B presents the distribution of treatment effects, defined as Δy=yACC=10−yACC=30.

As shown in Fig. 4A, increasing ACC from 10 to 30 mg/L consistently shifts the predicted distributions leftward across all temperatures. At 4°C/168 h, the peak decreases from 6.8 to 5.3 log CFU/g (a reduction of 1.5 log units). At 14°C/72 h, the peak shifts from 7.3 to 5.4 log CFU/g (a reduction of 1.9 log units). At 24°C/48 h, the peak decreases from 7.1 to 5.7 log CFU/g (a reduction of 1.4 log units). These reductions quantify the antimicrobial efficacy of higher ACC under different storage conditions.

Fig. 4B shows that the mean Δy values at 4, 14, and 24°C are 1.44, 1.88, and 1.36 log CFU/g, respectively. Marginal benefits peak at 14°C, followed by 4°C, and are lowest at 24°C. This non-monotonic temperature dependence can be explained by SAEW inhibition mechanisms: at low temperature (4°C), extended repair times for sublethal injury are offset by reduced metabolic activity of surviving cells; at 14°C, partial metabolic recovery allows ACC increases to effectively suppress growth initiation, maximizing marginal benefit; at 24°C, rapid proliferation dominates and sustained inhibition weakens (Liu et al., 2019; Xuan et al., 2017).

#### Superiority probability heatmap of SAEW treatment

Fig. 4C presents a superiority probability heatmap expressing the advantage of high ACC (30 mg/L) over low ACC (10 mg/L) as a function of temperature (*T*) and time (*t*). The color scale represents *P* (*T, t*), with values approaching yellow (0.8 – 1.0) indicating high model confidence that higher ACC yields lower bacterial counts.

As shown in Fig. 4C, the distribution of superiority probability exhibits distinct temperature-dependent patterns: At 4°C, the yellow high-confidence region spans the entire storage period (0 – 350 h). At 14°C, the high-confidence window extends across 0 – 130 h. At 24°C, high-confidence values (*p* > 0.8) appear only within the first 90 h. These findings indicate that, at 4°C, higher ACC (30 mg/L) maintains probabilistic dominance throughout a 7‑day storage period; under short‑term temperature abuse at 14°C, probabilistic advantages of higher ACC are mainly observed within the first 130 h; whereas at 24°C, the probabilistic benefit of SAEW diminishes rapidly relative to temperature control.

These scenario-based conclusions align closely with experimental findings reported by Mansur et al. (2015) and Sheng et al. (2018), suggesting that the hybrid model developed here has substantial potential for practical application.

## Conclusion

This study developed a physics‑informed hybrid machine learning framework to predict the growth of *S.* Enteritidis on chicken surfaces. The SGompertz model provided the mechanistic baseline, and CatBoost served as a residual corrector. SHAP analyses showed that residual learning effectively compensated for structural limitations of the mechanistic model and captured nonlinear thermal and antimicrobial effects. Scenario simulations further revealed a clear temperature dependence in the efficacy of SAEW and identified storage conditions under which higher ‑ ACC SAEW shows consistent probabilistic advantage: 4°C for up to 350 h, 14°C for up to 130 h, and 24°C for up to 90 h.

To translate this framework into practice, the superiority probability heatmaps can be converted into user‑friendly look‑up tables or web‑based calculators for rapid assessment by cold‑chain managers. The probability threshold of 0.8 provides a simple risk‑based rule for determining when higher ACC is warranted.

Limitations of this study include the reliance on constant‑temperature data for model training. Future work should examine performance under dynamic temperature fluctuations.

Overall, this framework provides an interpretable approach for diagnosing and correcting bias in mechanistic antimicrobial models and supports more precise management of isothermal cold-chain storage processes.

## Author Contributions

Yunxiang Zhu and Shan Bing conceived and designed the study, developed the physics-informed machine-learning framework, analyzed the data, interpreted the results, and drafted the manuscript. Yitian Zang contributed to the study design, interpretation of the findings, and substantial intellectual revision of the manuscript. Yanjiao Li, Guoping Wu, and Nengshui Ding contributed to the experimental design, conducted the microbiological experiments, and interpreted the results. Guosheng Zhang and Lili Xiao developed the analytical strategy, performed the statistical analyses, and interpreted the findings. Guoyun Wu, Zhen Wang, and Ruilin Wang contributed to the study design, interpretation of the results, and substantial intellectual revision of the manuscript. Fule Yang contributed to the acquisition and quality assessment of the experimental data and interpretation of the findings. Yucheng Zhang contributed to the experimental design, selection of experimental materials, and interpretation of the findings. Chenxi Cui, Yuhang Ma, and Hongxin Fu designed and conducted the model-validation procedures, assessed model robustness, and interpreted the validation results. All authors critically revised the manuscript, approved the final version, and agree to be accountable for the accuracy and integrity of the work.

## CRediT authorship contribution statement

**Yunxiang Zhu:** Writing – original draft. **Shan Bing:** Writing – original draft. **Yitian Zang:** Writing – review & editing, Funding acquisition. **Yanjiao Li:** Investigation. **Guoping Wu:** Investigation. **Guosheng Zhang:** Formal analysis, Data curation. **Nengshui Ding:** Investigation. **Guoyun Wu:** Supervision, Data curation. **Zhen Wang:** Supervision, Data curation. **Ruilin Wang:** Supervision, Data curation. **Fule Yang:** Data curation. **Yucheng Zhang:** Resources. **Lili Xiao:** Formal analysis. **Chenxi Cui:** Validation. **Yuhang Ma:** Validation. **Hongxin Fu:** Validation.

## Disclosures

The authors declare that they have no known competing financial interests or personal relationships that could have appeared to influence the work reported in this paper.

## References

[bib0001] Al‑Holy M.A., Rasco B.A (2015). The bactericidal activity of acidic electrolyzed oxidizing water against Escherichia coli O157:H7, salmonella typhimurium, and listeria monocytogenes on raw fish, chicken and beef surfaces. Food Control..

[bib0002] Baek S.-H., Lim C.-G., Park J.-I., Seo Y.-B., Nam I.-S. (2024). Investigation of *Salmonella Enteritidis* growth under varying temperature conditions in liquid whole egg: proposals for smart management technology for safe refrigerated storage. Foods.

[bib0004] Casanova C.F., de Souza M.A., Fisher B., Colet R., Marchesi C.M., Zeni J., Cansian R.L., Backes G.T., Steffens C. (2022). Bacterial growth in chicken breast fillet submitted to temperature abuse conditions. Food Sci., Technol..

[bib0005] Gibson A.M., Bratchell N., Roberts T.A. (1987). The effect of sodium chloride and temperature on the rate and extent of growth of *Clostridium botulinum* type A in pasteurized pork slurry. J. Appl. Bacteriol..

[bib0006] Guillén S., Domínguez L., Mañas P., Álvarez I., Carrasco E., Cebrián G. (2024). Modelling the low temperature growth boundaries of *Salmonella Enteritidis* in raw and pasteurized egg yolk, egg white and liquid whole egg: Influence of the initial concentration. Int. J. Food Microbiol..

[bib0008] Jia Z., Peng Y., Yan X., Zhang Z., Fang T., Li C. (2020). One-step kinetic analysis of competitive growth of *Salmonella* spp. and background flora in ground chicken. Food Control..

[bib0009] Kong D., Quan C., Xi Q., Han R., Koseki S., Li P., Du Q., Yang Y., Forghani F., Wang J. (2022). Study on the quality and myofibrillar protein structure of chicken breasts during thawing of ultrasound-assisted slightly acidic electrolyzed water (SAEW). Ultrason. Sonochem..

[bib0010] Kong D., Han R., Yuan M., Xi Q., Du Q., Li P., Yang Y., Wang J. (2023). Effects of ultrasound-assisted slightly acidic electrolyzed water thawing on myofibrillar protein conformation and gel properties of chicken breasts. Food Chem..

[bib0012] Li M., Huang L., Yuan Q. (2017). Growth and survival of *Salmonella Paratyphi* A in roasted marinated chicken during refrigerated storage: Effect of temperature abuse and computer simulation for cold chain management. Food Control..

[bib0013] Li Q., Chen S., Han J., Li B., Wu L., Li J. (2025). Unraveling almonds deterioration using whole-cell biosensor coupled with machine learning approaches and SHAP interpretation. Food Chem..

[bib0014] Liu Q., Jin X., Feng X., Yang H., Fu C. (2019). Inactivation kinetics of *Escherichia coli* O157:H7 and *Salmonella Typhimurium* on organic carrot (*Daucus carota* L.) treated with low concentration electrolyzed water combined with short-time heat treatment. Food Control..

[bib0016] Mansur A.R., Tango C.N., Kim G.-H., Oh D.H. (2015). Combined effects of slightly acidic electrolyzed water and fumaric acid on the reduction of foodborne pathogens and shelf life extension of fresh pork. Food Control..

[bib0017] Odeyemi O.A., Alegbeleye O.O., Strateva M., Stratev D. (2020). Understanding spoilage microbial community and spoilage mechanisms in foods of animal origin. Compr. Rev. Food Sci. Food Saf..

[bib0018] Oscar T.P. (2007). Predictive models for growth of *Salmonella typhimurium* DT104 from low and high initial density on ground chicken with a natural microflora. Food Microbiol..

[bib0019] Possas A., Posada-Izquierdo G.D., Pérez-Rodríguez F., Valero A., García-Gimeno R.M., Duarte M.C.T. (2016). Application of predictive models to assess the influence of thyme essential oil on *Salmonella Enteritidis* behaviour during shelf life of ready-to-eat turkey products. Int. J. Food Microbiol..

[bib0020] Paganini C.C., Longhi D.A., de Aragão G.M.F., Carciofi B.A.M. (2022). Modelling the inactivation, survival and growth of *Salmonella enterica* under osmotic stress considering inoculum phase and serotype. J. Appl. Microbiol..

[bib0021] Raissi M., Perdikaris P., Karniadakis G.E. (2019). Physics-informed neural networks: a deep learning framework for solving forward and inverse problems involving nonlinear partial differential equations. J. Comput. Phys..

[bib0022] Rahman S.M.E., Khan I., Oh D.H. (2016). Electrolyzed water as a novel sanitizer in the food industry: current trends and future perspectives. Compr. Rev. Food Sci. Food Saf..

[bib0023] Sheng X., Shu D., Tang X., Zang Y. (2018). Effects of slightly acidic electrolyzed water on the microbial quality and shelf life extension of beef during refrigeration. Food Sci. Nutr..

[bib0024] Thames H.T., Theradiyil Sukumaran A. (2020). A review of *Salmonella* and *campylobacter* in broiler meat: emerging challenges and food safety measures. Foods.

[bib0025] Willard J., Jia X., Xu S., Steinbach M., Kumar V. (2023). Integrating scientific knowledge with machine learning for engineering and environmental systems. ACM Comput. Surv..

[bib0026] Xuan X.-T., Ding T., Li J., Ahn J.-H., Zhao Y., Chen S.-G., Ye X.-Q., Liu D.-H. (2017). Estimation of growth parameters of *Listeria monocytogenes* after sublethal heat and slightly acidic electrolyzed water (SAEW) treatment. Food Control..

[bib0027] Yuan X., Li Y., Mo Q., Zhang B., Shu D., Sun L., Zhao X., Zhang R., Zheng J., Jia Y., Zang Y. (2023). Antibacterial activity and mechanism of slightly acidic electrolyzed water combined with ultraviolet light against *Salmonella Enteritidis*. Food Control..

[bib0028] Zang Y.T., Bing Sh., Li Y.J., Shu D.Q., Huang A.M., Wu H.X., Lan L.T., Wu H.D. (2019). Efficacy of slightly acidic electrolyzed water on the microbial safety and shelf life of shelled eggs. Poult., Sci..

[bib0029] Zang Y., Zang Y., Zhang Q., Zhang G., Hu J., Liu R., Tu M., Qiao W., Hu M., Fu B., Shu D., Li Y., Zhao X. (2024). Construction of a tertiary model and uncertainty analysis for the effect of time, temperature, available chlorine concentration of slightly acidic electrolyzed water on *Salmonella Enteritidis* and background total bacteria counts on chicken. LWT.

